# Qizhi Jieyu Pill for the Treatment of Cerebral Infarction During the Recovery Phase: Protocol for a Randomized Controlled Clinical Trial

**DOI:** 10.2196/75982

**Published:** 2025-08-13

**Authors:** Xuedong An, Liangqing Yu, Yuqin Ma, Junwei Zhang, Shiping Guo, Jilong Xu, Ping Zhao, Hongyan Xu, Feng Gao, Xueqin Wu, Yanlan Gao, Yao Jiang, Yali Liu

**Affiliations:** 1 Guang'anmen Hospital of China Academy of Chinese Medical Sciences Beijing China; 2 Wuzhai County Hospital of Traditional Chinese Medicine Xinzhou China

**Keywords:** cerebral infarction, recovery phase, Qizhi Jieyu pill, clinical research, study protocol

## Abstract

**Background:**

Cerebral infarction, a life-threatening neurodegenerative disease, is one of the leading causes of disability and death. The recovery phase is a critical period for patients to regain cognitive ability, memory, and motor functions and to improve their ability to perform daily activities. Over the past 10 years, we have applied Qizhi Jieyu pill in the treatment of patients in the recovery phase of cerebral infarction. We retrospectively analyzed clinical data from 152 patients and found preliminary results suggesting that Qizhi Jieyu pill may help alleviate clinical symptoms, improve quality of life, and reduce the recurrence of cerebral infarction. However, the lack of high-quality clinical evidence supporting its efficacy and safety has limited its widespread clinical application.

**Objective:**

This protocol describes a randomized controlled trial aiming to evaluate the efficacy and safety of Qizhi Jieyu pill in patients during the recovery phase of cerebral infarction.

**Methods:**

This study adopts a multicenter, randomized controlled clinical trial design. A total of 126 patients aged 30-70 years in the recovery phase of cerebral infarction will be recruited and randomly assigned in a 1:1 ratio to either the study group (n=63, receiving conventional treatment plus Qizhi Jieyu pill) or the control group (n=63, receiving conventional treatment alone). The intervention period will last 12 weeks, and the total follow-up period will be 48 weeks. The primary outcome is the change in the National Institutes of Health Stroke Scale score at week 48. Secondary outcomes include changes in traditional Chinese medicine syndrome scores, the recurrence rate of cerebral infarction, and modified Rankin Scale scores. Adverse events will be recorded and used to assess the safety profile. All data analyses will be performed according to a prespecified statistical analysis plan.

**Results:**

The study received ethics approval on March 3, 2025, from the Ethics Committee of Guang’anmen Hospital, China Academy of Chinese Medical Sciences (2025-025-KY-01) and was registered on April 6, 2025. Participant recruitment began on April 10, 2025, and is expected to be completed by December 31, 2025. Final results will be published by December 31, 2026.

**Conclusions:**

This study is the first randomized controlled clinical trial to evaluate the efficacy and safety of Qizhi Jieyu pill in the treatment of patients during the recovery phase of cerebral infarction. It will contribute to the development of integrated treatment strategies, potentially leading to broader clinical adoption, by providing a high-quality, evidence-based treatment option for clinical practice.

**Trial Registration:**

International Traditional Medicine Clinical Trial Registry ITMCTR2025000653; https://itmctr.ccebtcm.org.cn/mgt/project/view/2028021950497347405/false

**International Registered Report Identifier (IRRID):**

PRR1-10.2196/75982

## Introduction

Cerebral infarction, a life-threatening neurodegenerative disease, predominantly affects older adults and is classified as an ischemic cerebrovascular event [1]. Due to the various degrees and types of brain damage it causes—such as brain tissue lesions, structural damage, neuronal death, and dysfunction—it remains one of the leading causes of disability and death [2,3]. According to the 2019 Global Burden of Disease study, the age-standardized incidence rate of cerebral infarction in China was 144 per 100,000, and the age-standardized prevalence was 1255.9 per 100,000. With the acceleration of population aging, the burden of this disease is expected to continue rising [4].

The acute phase of cerebral infarction progresses rapidly, with most patients entering the recovery phase approximately 2 weeks after onset, which typically lasts about 6 months. The recovery phase is crucial for regaining cognitive ability, memory, and motor functions, thereby enhancing daily living capabilities. Hence, rehabilitation during this stage is a hot topic in clinical research [5]. Core treatment strategies during this phase primarily include antiplatelet therapies, such as aspirin, clopidogrel, sarpogrelate, and cilostazol, aimed at reducing stroke recurrence and improving neurological function [6-8]. However, these treatments have yet to effectively address the high recurrence and disability rates.

Multiple clinical studies have confirmed that traditional Chinese medicine (TCM) therapies—such as Buyang Huanwu decoction and acupuncture—can significantly improve clinical symptoms during the recovery phase of cerebral infarction. These include reducing the recurrence rate of cerebral infarction, enhancing neurological function, and mitigating the severity of neurological deficits, making TCM a vital component of stroke rehabilitation [9-14].

We have used Qizhi Jieyu pill, composed of traditional herbs such as *Panax notoginseng* and leech, in clinical practice for over 10 years to treat patients during the recovery phase of cerebral infarction. Preliminary, retrospective analysis of 152 cases indicates that Qizhi Jieyu pill may alleviate clinical symptoms, improve quality of life, and reduce recurrence. This suggests its potential as an important therapeutic option during the recovery phase. However, there is still a lack of high-quality clinical studies to validate its efficacy and safety.

This study aims to evaluate the efficacy and safety of Qizhi Jieyu pill in the treatment of patients during the recovery phase of cerebral infarction through a randomized controlled clinical trial. We hypothesize that the addition of Qizhi Jieyu pill to conventional treatment will result in greater improvement in National Institutes of Health Stroke Scale (NIHSS) scores and lower recurrence rates compared to conventional treatment alone.

## Methods

### Study Design

This study aims to evaluate the efficacy and safety of Qizhi Jieyu pill in the treatment of cerebral infarction during the recovery phase. It adopts a multicenter, randomized controlled clinical trial design and will be conducted in accordance with the principles of the Declaration of Helsinki, the Good Clinical Practice guidelines, and Chinese regulations. The study will be carried out at Guang’anmen Hospital, China Academy of Chinese Medical Sciences, and Wuzhai County Hospital of Traditional Chinese Medicine.

### Ethical Considerations

The Ethics Committee of Guang’anmen Hospital of the China Academy of Chinese Medical Sciences approved the study (2025-025-KY-01). All patients provided their written informed consent to participate in the study and authorized the publication of their data. The trial has been registered with the International Traditional Medicine Clinical Trial Registry (ITMCTR2025000653).

### Sample Size Calculation

The clinical sample size was calculated using a 2-group mean difference test (shown below), where n is the required sample size per group, σ is the pooled SD of the 2 groups, α is the type 1 error, β is the type 2 error, and δ is the expected mean difference between the study and control groups.




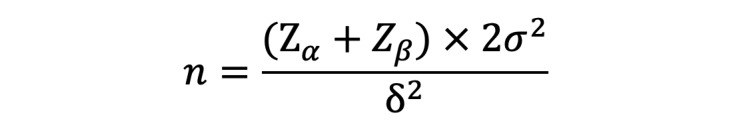




**Figure 1:**
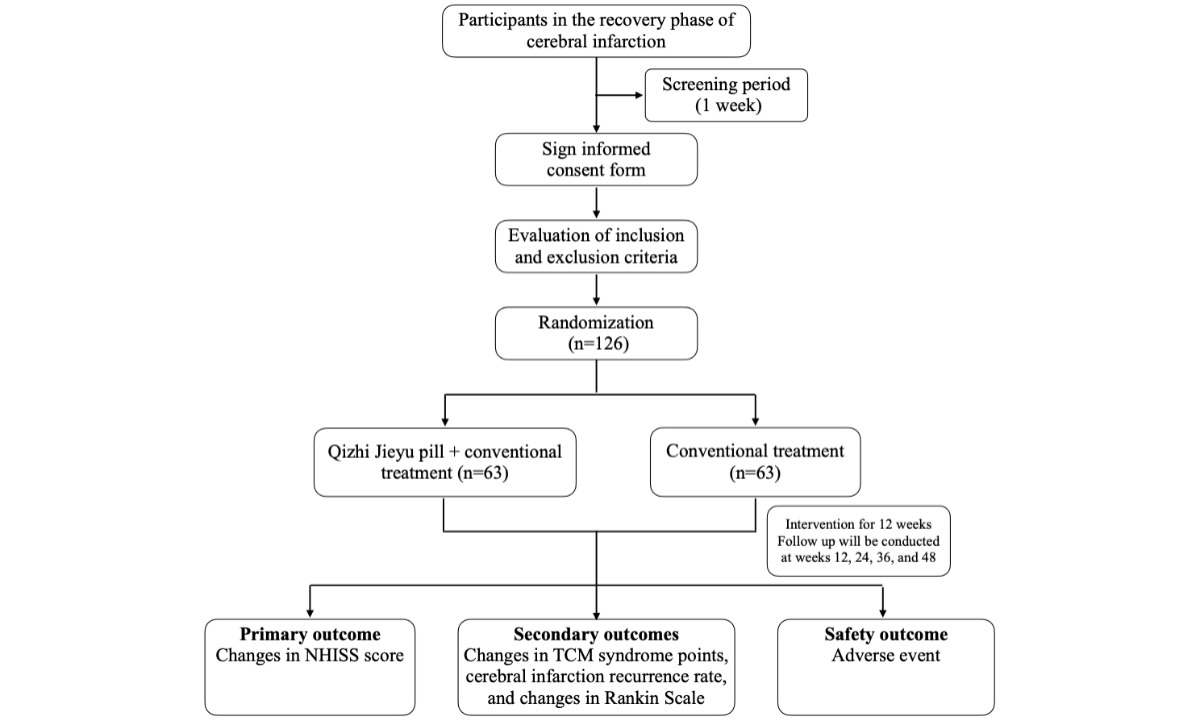
Study flowchart illustrating the trial design, including the screening of 126 eligible participants with cerebral infarction in the recovery phase, randomization into 2 groups (Qizhi Jieyu pill plus conventional treatment versus conventional treatment alone), a 12-week treatment period, and follow-up assessments at weeks 12, 24, 36, and 48. The outcomes assessed include changes in National Institutes of Health Stroke Scale (NIHSS) scores, traditional Chinese medicine (TCM) symptom scores, recurrence rates, Rankin Scale scores, and adverse events.

Since there are currently no direct data available to calculate the clinical sample size, we mainly referred to 2 clinical studies [15,16]. In these studies, the NIHSS score change value in the control group (conventional treatment) was approximately 4.9. The pooled SD (σ) was estimated based on prior studies that reported SDs ranging from 1.5 to 2.5. We conservatively used σ=2.0 in our calculation to ensure adequate power. It is hypothesized that the change in the study group (conventional treatment plus Qizhi Jieyu pills) would be approximately 6, with a mean difference of 2 between the 2 groups. Assuming a 1:1 sample ratio between the control and study groups with α=0.05 and β=0.2, the required sample size per group is 52. Considering an overall dropout rate of 20%, each group needs 63 participants, for a total of 126 participants. A third-party group composed of experts in TCM, endocrinology, and statistics will be organized at the midterm of the study to reassess the sample size based on the observed effect size of the primary efficacy indicators and associated errors. Figure 1 shows the study flowchart.

### Participants

#### Cerebral Infarction Diagnostic Criteria

Referring to the 2014 Chinese guidelines for diagnosis and treatment of acute cerebral infarction issued by the Cerebrovascular Disease Group of the Neurology Branch of the Chinese Medical Association [17], diagnostic criteria include sudden onset, focal neurological deficits (occasionally generalized), symptoms and signs lasting more than 24 hours, exclusion of nonvascular brain lesions, and computed tomography (CT) or magnetic resonance imagining (MRI) excluding cerebral hemorrhage or other abnormalities, with a responsible ischemic lesion.

#### Diagnostic Criteria for the Recovery Phase of Cerebral Infarction

Referring to the 2017 guidelines for integrated traditional Chinese and western medicine diagnosis and treatment of cerebral infarction in China [18], within 6 hours of onset is the hyperacute phase, around 2 weeks postonset is the acute phase, 2 weeks to 6 months postonset is the recovery period, and more than 6 months postonset is the sequelae phase.

#### Diagnostic Criteria for Phlegm-Stasis Obstruction Syndrome

The diagnostic criteria are based on the Standards for the TCM Diagnosis and Therapeutic Evaluation of Stroke (Trial Version, 1995), drafted by the Encephalopathy Emergency Research Collaboration Group of the State Administration of Traditional Chinese Medicine [19], and include dizziness and vertigo, profuse and sticky phlegm, dark tongue body, thin white or greasy white tongue coating, and a wiry-slippery pulse.

#### Inclusion Criteria

Criteria for inclusion in the study are as follows:

1. Patients diagnosed with cerebral infarction and currently in the recovery phase.
2. Patients aged 30-70 years.
3. Patients who voluntarily signed the informed consent form.

#### Exclusion Criteria

Criteria for exclusion from the study include the following:

1. Patients diagnosed with intracranial hemorrhagic disease by head CT or MRI.
2. Patients with abnormal clinical laboratory tests, including alanine aminotransferase or aspartate aminotransferase ≥2.5 times the upper limit of normal or creatinine above the upper limit of normal.
3. Patients unable to understand or comply with the study protocol due to severe psychiatric, cognitive, or emotional disorders.
4. Patients with other diseases affecting limb mobility that may impact neurological assessments.
5. Women who are pregnant, breastfeeding, or planning to conceive in the near future.
6. Patients with a suspected or confirmed history of alcohol or drug abuse within 6 months prior to onset.
7. Patients with a known or suspected allergy to the investigational drug or those with an allergic constitution.
8. Patients who have participated in another clinical trial within the last 3 months.
9. Other situations judged by the investigator to reduce the likelihood of enrolment or complicate enrolment.

#### Criteria for Case Elimination

The following are criteria for case elimination:

1. After enrollment, cases were found not to meet the inclusion criteria or to meet the exclusion criteria.
2. Cases involving the use of prohibited medications as per protocol, necessitating elimination due to protocol violations.
3. Cases in which the medication was never received or the exposure did not meet the experimental requirements.
4. Cases with no evaluable records after medication administration.

#### Trial Termination Criteria

Satisfaction of any of the following criteria will result in discontinuation of the clinical trial before the planned end in order to protect participants’ rights and ensure research quality:

1. Serious safety issues arising during the study requiring immediate termination.
2. Major errors discovered in the clinical research protocol, making it difficult to evaluate drug efficacy; or important deviations occurring from a well-designed protocol, compromising the efficacy evaluation.
3. Very favorable outcomes observed in the TCM group during the study warranting that the trial be terminated early to allow the control group to receive the TCM treatment.
4. Termination requested by the research team (eg, due to funding or management issues).
5. Termination required by administrative authorities.

#### Conditions and Procedures for Participant Withdrawal

There are two routes for participant withdrawal.

Withdrawal determined by the investigator: This may occur when a participant already enrolled in the study becomes unsuitable for continued participation due to the development of complications, comorbidities, or special physiological changes. Other conditions include poor compliance, defined as taking less than 80% or more than 120% of the prescribed medication, and the use of prohibited medications as per protocol.

Participant-initiated withdrawal: According to the informed consent form, participants have the right to withdraw at any time. If a participant stops taking the medication or attending visits without formally withdrawing, they are still considered “withdrawn” (or “lost to follow-up”). Reasons for withdrawal should be documented where possible and may include feeling the treatment is ineffective, the development of intolerable adverse reactions, personal circumstances, financial reasons, or unexplained loss to follow-up.

Regardless of the reason, case report forms for withdrawn participants should be retained. The last recorded test results will be used as final data, and both efficacy and adverse events will be analyzed in the full dataset.

### Intervention

Referring to the guidelines for clinical management of cerebrovascular diseases in China (second edition) [20], both groups will receive conventional rehabilitation training guidance, including assessment and rehabilitation of cognitive function, speech ability, swallowing function, limb motor and sensory function, balance, psychological state, and daily living abilities. They will also receive standard treatments such as antiplatelet therapy, lipid-lowering and blood pressure control, plaque stabilization, and electrolyte balance maintenance.

On the basis of conventional treatment, the study group will take Qizhi Jieyu pills, 9 g per dose, twice daily after meals. The control group will receive conventional treatment only. The intervention period is 12 weeks, with a total follow-up period of 48 weeks.

All TCM drugs with the same therapeutic effect as the investigational drug (including those with similar indications or efficacy stated in the instructions) are prohibited during the study. All concomitant medications and treatments (ie, for other diseases) must be recorded in the concomitant medication log.

### Study Procedures

Table 1 outlines the specific measurements and timepoints for data collection in this study. Eligible participants will undergo a 1-week preintervention screening period, a 12-week treatment period, and a total 48-week follow-up period. After the study begins, all enrolled participants will have follow-up visits every 12 weeks. All participants will be informed of the study through oral and written information and will be required to sign an informed consent form.

**Table 1:** Clinical trial research timeline.

Item		Screening period and baseline	Visit 1	Visit 2	Visit 3	Visit 4
Visit time		–7 to 0 days	12 weeks ± 7 days	24 weeks ± 7 days	36 weeks ± 7 days	48 weeks ± 7 days
**Collect basic medical history**						
	Sign informed consent form	×				
	Fill in general information	×				
	Medical history and treatment history	×				
	Determine the inclusion and exclusion criteria	×				
	Vital signs	×	×	×	×	×
	Physical examination	×	×	×	×	×
	Merge diseases and medication	×	×	×	×	×
**Diagnosis and monitoring inspection**						
	Urine pregnancy test	×				
	Blood sugar, blood pressure, and blood lipids	×	×	×	×	×
	Brain CT^a^ or MRI^b^	×	×	×	×	×
**Effectiveness outcome**						
	NHISS^c^ score	×	×	×	×	×
	Recurrent cerebral infarction	×	×	×	×	×
	Traditional Chinese medicine symptom score	×	×	×	×	×
	Rankin Scale	×	×	×	×	×
**Physical and chemical examination**						
	Routine bloods, routine urine, and microscopic examination	×			×	×
	Routine stool	×			×	×
	Vital signs	×	×	×	×	×
	Electrocardiogram and liver and kidney function	×	×	×	×	×
	Adverse events		×	×	×	×
**Other items**						
	Randomization	×				
	Distribute medication and patient diary cards	×	×	×	×	
	Recycling drugs and quantity statistics		×	×	×	×
	Retrieve patient diary cards		×	×	×	×
	Conclusion of the study					×

^a^CT: computed tomography.

^b^MRI: magnetic resonance imaging.

^c^NIHSS: National Institutes of Health Stroke Score.

### Outcomes

#### Primary Outcome

The primary outcome is the change in NIHSS score at 48 weeks [21]. This scale evaluates levels of consciousness, command compliance, eye movement, visual field defects, facial paralysis, limb motor dysfunction, ataxia, speech expression, and other parameters.

#### Secondary Outcomes

The 3 secondary outcomes are the change in TCM syndrome scores, cerebral infarction recurrence rates, and the change in Rankin Scale scores.

A comparison of TCM syndrome scores before and after the medication will be conducted. Based on the guiding principles for clinical research of new Chinese medicines [22], efficacy is categorized as cured, markedly effective, effective, or ineffective according to the change in TCM syndrome scores. TCM symptoms include dizziness, headache, irritability, limb stiffness, neck stiffness, limb numbness, excessive phlegm, shortness of breath, fatigue, spontaneous sweating, dry stool or constipation, dry mouth or thirst, red or dark tongue, and yellow-greasy tongue coating.

In addition, we will use an intelligent TCM diagnostic device (produced by Shenzhen Huiyibida Medical Technology Co, Ltd) to collect tongue images, tongue coating, and pulse information from patients at multiple timepoints. Two study physicians will independently record patients’ clinical symptom information to monitor changes in TCM symptoms and the potential evolution of syndromes.

The cerebral infarction recurrence rate will be evaluated every 12 weeks by CT or MRI and calculated as the number of recurrent cases / the total study population × 100%.

Finally, a comparison of pretreatment and post-treatment Rankin Scale scores will be conducted [23]. The Rankin Scale evaluates neurological recovery after stroke in 6 levels: 0=no symptoms at all, 1=symptoms present but no significant disability, 2=slight disability, 3=moderate disability, 4=moderately severe disability, 5=severe disability, and 6=death.

#### Safety Outcomes

Safety will be assessed using the following monitoring indicators:

1. Vital signs, including temperature, blood pressure, respiration rate, and heart rate, checked every 12 weeks.
2. Routine tests, including blood, urine, and stool tests, every 12 weeks.
3. Other labs and diagnostics, including electrocardiograms, liver function tests (alanine aminotransferase, aspartate aminotransferase, gamma-glutamyltransferase, alkaline phosphatase, and total bilirubin), and kidney function tests (blood urea nitrogen and creatine), every 12 weeks.
4. Adverse events: From the time the participant enters the study until the final follow-up, adverse events will be monitored for 48 weeks. All information related to adverse events will be carefully recorded at all times. Serious adverse events refer to any unexpected clinical events occurring at any dose that result in hospitalization, prolongation of existing hospitalization, disability, impaired ability to work, life-threatening conditions or death, or congenital malformations. Serious adverse events will be reported to the study center and the principal medical ethics committee. In addition, they will be reported to the National Medical Products Administration within 24 hours. Meanwhile, we will specifically record adverse events potentially related to animal-derived components, such as itching, rash, allergic reactions, and gastrointestinal discomfort.

### Randomization

Random numbers will be generated using SAS statistical software (version 9.4), and participants will be assigned to the intervention and control groups in a 1:1 ratio. After screening and informed consent, patients will be randomly assigned to one of the two groups based on their order of enrolment. The drug number for each participant will remain unchanged throughout the study. In the case of a clinical emergency, the participant’s random code and group assignment may be unblinded.

Due to the nature of the intervention, participant and clinician blinding is not feasible. However, outcome assessors will be blinded to group assignments to minimize detection bias, especially for subjective measures such as TCM symptom scores.

### Data Collection and Management

#### Clinical Materials and Samples

All materials, clinical test results, and biological samples (blood, urine, and stool) will be stored securely at each subcenter after every follow-up and handed over to the principal investigator at the end of the study for archiving.

#### Data Recording and Storage Method

Paper-based case report forms (CRFs) will be used for this study. Although paper-based CRFs will be used, we will adopt a double-entry system and periodic data verification procedures. Digital scans of CRFs will be backed up, and a central data management team will oversee consistency and integrity across sites.

#### Data Quality Assurance Measures

Standard operating procedures will be established. All study personnel, including clinical investigators and CT and MRI technicians, will receive unified training.

All principal investigators at participating centers will be assessed and authorized by the project leader.

Requirements for filling in the CRFs will be strictly enforced. Records must be accurate, complete, and truthful. Corrections, if necessary, should be made with a single line through the error. The correction should be entered next to it, signed and dated.

Monitoring and audit standard operating procedures will be developed. Regular on-site monitoring visits will be conducted by the project leader to ensure compliance with the study protocol and verify that CRF data match the original source documents.

### Statistical Analysis

#### Definition and Selection of Statistical Analysis Datasets

Participants will be categorized into the following analysis sets:

1. Full analysis set: This includes all randomized participants who have at least 1 postbaseline efficacy evaluation. This is the main dataset for efficacy analysis. Missing values for primary efficacy outcomes will be imputed using the last observation carried forward method. Missing values for secondary efficacy and safety outcomes will not be imputed. For secondary efficacy endpoints and safety data, descriptive analysis and complete-case analysis will be conducted based on actual observed values. Although last observation carried forward is commonly used in clinical trials, we acknowledge its limitations in progressive conditions such as stroke recovery. We will perform sensitivity analyses using multiple imputation methods to evaluate the robustness of our results.
2. Per protocol set: This includes all participants in the full analysis set who meet inclusion criteria, do not meet exclusion criteria, complete the treatment, and do not have major protocol deviations. This is the secondary dataset for efficacy evaluation.
3. Safety set: This includes all randomized participants who received at least 1 dose of Qizhi Jieyu pill and had at least 1 postbaseline safety evaluation.

#### Statistical Analysis Plan

A statistical analysis plan was formulated prior to the start of the study. It will be finalized after discussion among the statisticians, principal investigators, and sponsor before database lock. This plan describes only the standard statistical requirements.

Statistical analysis will be conducted using SAS version 9.4. All hypothesis tests will be 2-sided, with a significance level of α=.05. *P* values less than or equal to .05 will be considered statistically significant.

For continuous variables, mean, SD, minimum, maximum, and median values will be used for descriptive statistics. Independent samples *t* tests (for normally distributed data) or Wilcoxon rank-sum tests (for non-normally distributed data) will be used for hypothesis testing. Normality of continuous variables will be assessed using the Shapiro-Wilk test before choosing appropriate parametric or nonparametric tests.

For categorical variables, frequency and percentages will be used for description. Chi-square tests or Fisher exact tests will be used for hypothesis testing. Ordinal variables will be analyzed using the Wilcoxon rank-sum test.

Logistic regression will be used to control for confounding factors affecting the outcome measures. We will also analyze the association between syndrome evolution patterns and therapeutic efficacy. In parallel, multivariate linear regression will be used to explore the relationship between outcomes and potential confounders, such as baseline NIHSS score, study center, age, and sex. For adverse events potentially associated with animal-derived components—including itching, rash, allergic reactions, and gastrointestinal discomfort—we will conduct attribution analysis and report their incidence separately.

## Results

The study was approved by the Ethics Committee of Guang’anmen Hospital, China Academy of Chinese Medical Sciences, on March 3, 2025 (2025-025-KY-01), and was registered on the International Traditional Medicine Clinical Trial Registry on April 6, 2025 (ITMCTR2025000653), and any potential protocol amendments will be recorded there. Participant screening began on April 10, 2025, with enrolment expected to be completed by December 31, 2025. The final study results are planned to be published by December 31, 2026.

This study aims to evaluate the efficacy and safety of Qizhi Jieyu pill in the treatment of patients in the recovery phase of cerebral infarction, providing a treatment option supported by high-quality, evidence-based medicine for the clinical management of recovery phase cerebral infarction.

## Discussion

### Principal Findings

Given the functional impairments and neurological damage caused by cerebral infarction, it remains one of the leading causes of mortality and disability. Qizhi Jieyu pill has been used in clinical practice for over 10 years, with its primary therapeutic advantages including the improvement of clinical symptoms and reduction in the recurrence rate of cerebral infarction. However, due to the lack of standardized clinical research, there is currently no high-quality, evidence-based support for its efficacy and safety, which limits its widespread clinical application. Therefore, conducting this study to evaluate its effectiveness and safety in the recovery phase of cerebral infarction is of significant clinical value.

The upcoming study adopts a multicenter, randomized controlled clinical trial design, with a rigorously calculated sample size. Given the therapeutic advantages of Qizhi Jieyu pill, we primarily include patients in the recovery phase, which is a critical period for exerting its therapeutic effects. For outcome evaluation, we considered the adverse outcomes caused by cerebral infarction, including disability rates. Thus, we selected the change in NIHSS scores as the primary outcome, and changes in TCM syndrome scores, the recurrence rate of cerebral infarction, and Rankin Scale scores as secondary outcomes. Our standardized design facilitates rigorous and normative evidence acquisition. While the NIHSS and Rankin Scale are internationally recognized neurological assessments, TCM syndrome scores reflect TCM’s holistic evaluation of patient recovery. By combining these, we aim to provide a comprehensive understanding of therapeutic efficacy from both biomedical and TCM perspectives.

Qizhi Jieyu pill consists of 26 herbal and animal-derived medicinal components, including *Panax notoginseng, Hirudo, Buthus martensii, Scolopendra, Pheretima, Gastrodia elata, Uncaria, Cassia seed, Bombyx batryticatus, Acorus tatarinowii, Polygala, Typhonium giganteum,* and *Astragalus*, among others. It is formulated to remove blood stasis and phlegm, unblock collaterals to extinguish wind, and tonify the spleen and kidneys. Astragaloside IV, a major active compound of *Astragalus*, may promote angiogenesis via the silencing regulatory protein 7 (SIRT7)/vascular endothelial growth factor A (VEGFA) signaling pathway to improve brain tissue damage postcerebral infarction [24]. Astragaloside I has been shown to activate the transforming growth factor-β (TGF-β)/Smad signaling pathway and exert antioxidant effects, significantly improving cognitive dysfunction in rats with cerebral infarction [25,26]. *Gastrodia elata* can effectively reduce brain necrosis area, infarct volume, and inflammatory factor levels in rats with cerebral ischemia-reperfusion injury, with mechanisms involving the modulation of gut microbiota [27]. In addition, gastrodin, an active ingredient in *Gastrodia elata*, can increase the expression of miR-20a-5p in circulating exosomes and X-linked inhibitor of apoptosis protein (XIAP) in the ischemic hemisphere, thus promoting neurological recovery in rats with middle cerebral artery occlusion [28]. Ginsenoside Rg1 (G-Rg1), a key component of *Panax ginseng*, can reduce neural injury, infarct volume, and blood-brain barrier permeability in focal cerebral ischemia in rats, potentially through downregulation of protease-activated receptor-1 (PAR-1) expression [29]. These studies provide mechanistic support for the potential of Qizhi Jieyu pill as an effective treatment for cerebral infarction.

### Comparison to Prior Work

Compared with prior clinical trials evaluating TCM interventions, such as Tiandan Tongluo capsule [16] and Yindan Xinnaotong soft capsule [15], for poststroke rehabilitation, our study exhibits several innovative features. First, it adopts a longer follow-up period of 48 weeks, enabling a more comprehensive assessment of long-term outcomes such as recurrence and disability. Second, it incorporates a broader inclusion framework without limiting to specific TCM syndrome types, thus improving generalizability. Third, the outcome evaluation uses a dual-system framework that combines biomedical scales (NIHSS and modified Rankin Scale) with standardized TCM syndrome scores, thus enhancing the clinical interpretability of integrative medicine. Finally, our trial is grounded in over a decade of practical experience with Qizhi Jieyu pill and is the first to systematically evaluate this compound through a rigorously designed randomized controlled trial supported by mechanistic insights from pharmacological research.

### Strengths and Limitations

This study is innovative as it is the first rigorously designed randomized controlled trial to evaluate Qizhi Jieyu pill in the recovery phase of cerebral infarction. It introduces an integrative outcome framework combining biomedical and TCM assessments, targets a critical therapeutic window often overlooked in prior studies, and adheres to international methodological standards to provide high-quality, generalizable evidence for clinical practice. However, this study has certain limitations in its design. First, we did not adopt a blinded design, primarily because the recovery phase represents a critical therapeutic window for patients with cerebral infarction during which timely and standardized treatment is essential. Second, while Qizhi Jieyu pill includes both herbal and animal-derived components and our evidence may demonstrate significant clinical benefits, the active ingredients and mechanisms of action will not be specifically explored in this study; we plan to investigate these in subsequent research. Furthermore, our inclusion and exclusion criteria excluded patients younger than 30 years as they represent a low-risk population for cerebral infarction and older than 70 years due to common comorbidities and lower adherence, which could impact the effectiveness and safety evaluation of our intervention.

### Future Directions

In summary, this is the first randomized controlled clinical trial evaluating the efficacy and safety of Qizhi Jieyu pill for patients in the recovery phase of cerebral infarction. The study is expected to contribute to the development of strategies for the clinical treatment of poststroke recovery and promote its successful application in routine clinical practice.

## Abbreviations

CRF: case report form
CT: computed tomography
MRI: magnetic resonance imaging
NIHSS: National Institutes of Health Stroke Scale
TCM: traditional Chinese medicine
